# Ethical and legal aspects in the care of singers and actors^☆^

**DOI:** 10.1016/j.bjorl.2017.08.005

**Published:** 2017-08-23

**Authors:** Gustavo Polacow Korn, Carlos Michaelis, Vania Rosa Moraes

**Affiliations:** a Universidade Federal de São Paulo (Unifesp/EPM), Escola Paulista de Medicina, São Paulo, SP, Brazil; b Universidade de Coimbra, Direito em Medicina, Coimbra, Portugal; c Escola Paulista de Direito (EPD), Direito Médico, São Paulo, SP, Brazil; d Associação Brasileira de Otorrinolaringologia e Cirurgia Cérvico-Facial (ABORL-CCF), Departamento Jurídico, São Paulo, SP, Brazil

When treating singers and actors, especially in the acute phase, many clinical questions arise, such as: Can he/she sing or act? Should he/she sing or act? Is a lesion already present? Is there risk of injury? Additionally, ethical and legal aspects also deserve consideration when treating these professionals.

Failure to appropriately respond to these issues may result in violations of the Medical Ethics Code and Federal Medical Council Resolutions. Therefore, it is appropriate to present some care guidance and recommendations for situations when these individuals seek treatment; this begins at the reception. The attitude of the establishment's staff is crucial. They should be instructed to treat famous people like all other patients and not to request autographs or take selfies with them. The singer or actor, when seeking medical assistance, is in a possible situation of vulnerability and needs to be protected. The posting of such selfie on social media or sending it by WhatsApp violates the principle of medical confidentiality; it is forbidden for a physician to refer to identifiable clinical cases, to display patients or their portraits in the general communication media, even with the patient's consent.

At the reception, in addition to the patient, family members, bodyguards, manager, producer, press officer and others may be present. It is important to ask the patient who should be allowed to enter the consultation room, who should accompany him/her during the anamnesis, physical examination, and discussion of diagnostic hypotheses and conduct, which can rarely result in suspension of performance.^1^ When speaking with the patient, it is essential to be careful with the words chosen and always to be supportive. Regarding the production, always remember the importance of confidentiality. Depending on the situation, it is advisable to ask non-family members to give the client privacy when presenting an unfavorable diagnosis.

In the case of a performance cancelation, it is essential to inform the patient about the risk of fibrosis or injury if he/she chooses to go through with the performance. Such information should be included in the patient's medical records. As recommended by the Federal Medical Council (CFM), the medical record must contain the clinical data necessary for adequate case management and it must be filled out, in chronological order, containing the date, time, doctor's signature and Regional Medical Council (CRM) registration number.^2^

Any information on the client must remain within the health facility. It should be noted that other people might attempt to obtain information about the client's health and career.^3^ When it is the patient who decides to publish pictures with the physician, the latter can decide whether to agree with it or not. The advice of the Federal Medical Council is that they do not allow it, as if it is found out that the doctor is being “highly praised” by patients, the CFM will investigate. Use of WhatsApp should also be limmited, private and confidential and cannot go beyond the limits of the closed group of experts, the clinical staff or between doctors and their patients. To providing consultations, making diagnoses or prescribing medications by any means of communication is still prohibited, under the penalties of the law, according to the Legal Department of ABORL-CCF which participates in this editorial.

Finally, clinical cases of professional use of the voice are of great interest to colleagues who work more in the larynx and voice area. In congresses, symposia and courses, many of the details of such cases are of great didactic relevance. The identity of the patients deserves complete confidentiality, and their disclosure should only occur upon written authorization by the patient or his or her legal guardian.^4^ Similarly, the lecturer should consider not authorizing the recording, filming or photographing the material because it can be misused by the event participants, for example, in social media.

## Conflicts of interest

The authors declare no conflicts of interest.

## References

[1] Mishra S., Rosen C.A., Murry T. (2000). 24 hours prior to curtain. J Voice.

[2] Brasil, Resolução CFM n° 1931, de 24 de setembro de 2009, aprova o Código de Ética Médica, Diário Oficial da União de 13 de outubro de 2009, Seção I, p. 90. Retificação publicada no Diário Oficial da União de 13 de outubro de 2009, Seção I, p. 173.

[3] Sataloff R.T., Benninger M.S., Benninger M.S., Murry T. (2006). The performer's voice.

[4] Brasil, Resolução CFM n° 1974, de 19 de agosto de 2011, Estabelece os critérios norteadores da propaganda médica, Diário Oficial da União de 19 de agosto de 2011, Seção I, p. 241–244.

